# Exploring the Narratives of Patients With Cancer Using Large Language Models: Topic Modeling and Social Network Analysis

**DOI:** 10.2196/92539

**Published:** 2026-07-06

**Authors:** Xinyu Feng, Hin Chi Kwok, Ching Kok Chung, Janelle Yorke, Vivian Hui

**Affiliations:** 1 School of Nursing, The Hong Kong Polytechnic University, 11 Yuk Choi Road, Hung Hom, Kowloon, China (Hong Kong), 852 27664691; 2 School of Nursing, University of Manchester, Manchester, United Kingdom; 3 Department of Health and Community Sytems, School of Nursing, University of Pittsburgh, Pittsburgh, PA, United States

**Keywords:** TopicGPT, topic modeling, large language model, cancer, psychosocial stressors, network analysis, text mining

## Abstract

**Background:**

Patients with cancer often experience diverse psychosocial stressors that profoundly affect disease trajectories, treatment adherence, and overall quality of life. Understanding how patients experience and articulate these issues is critical for designing patient-centered interventions. Conventional data collection methods, such as surveys and interviews, provide depth but are constrained by recall bias and scalability and may overlook sensitive or underreported concerns. Patient-authored narratives in online health communities present a valuable opportunity to identify prevalent and underserved issues. However, critical analytic challenges remain in generating coherent and interpretable insights due to their unstructured and large-scale nature.

**Objective:**

This study aims to leverage TopicGPT, a prompt-based topic modeling framework powered by large language models (LLMs), in combination with network analysis for interpretable topic discovery and interrelationship analysis in the narratives of patients with cancer.

**Methods:**

Patient-authored posts describing psychosocial challenges about cancer experience were collected from 4 online health communities. Eligible posts were preprocessed and analyzed using TopicGPT, wherein topics were generated hierarchically and mapped at the sentence level. Comparison analyses were conducted among 3 state-of-the-art LLMs through cosine similarity and manual evaluation. Results from the best-performing LLM were further compared with 2 conventional topic models through topic diversity and were used to construct the network subsequently. Topic co-occurrence was examined using the pointwise mutual information algorithm and centrality metrics to reveal influential topics and thematic interconnections across narratives.

**Results:**

A total of 11,306 posts were collected from Reddit, Macmillan, Mijian, and Douban between December 6, 2006, and September 24, 2025. Of these, 3169 posts were retained for topic modeling and network analysis. DeepSeek-V3.2 consistently outperformed Gemini-2.5-Flash and GPT-4o, with similarity scores of 0.6295, 0.5342, and 0.5247, respectively. TopicGPT maintained consistently high topic diversity across languages. “Fear of cancer recurrence” and “Psychological distress” emerged as both most frequent and bridging topics across a hierarchy comprising 42 top-level and 58 subtopics. Strong connections were observed among “Sexual health concerns,” “Reproductive concerns,” and “Quality of life impact”; “Family communication concerns” frequently co-occurred with “Employment concerns,” “Diagnostic delays and misdiagnosis,” and “Social support.”

**Conclusions:**

This study demonstrates the potential of LLM-based topic modeling for large-scale, context-sensitive analysis of patient-authored narratives. The proposed integrated, domain-adaptable pipeline enables the identification of high-fidelity topics and their interrelationships, offering a scalable and interpretable approach to qualitative data in health care. Importantly, our findings reveal substantial concerns and unmet needs among patients with cancer, with potential to support patient-centered research and inform future clinical assessment and supportive care strategies.

## Introduction

According to the latest statistics from the International Agency for Research on Cancer, the global cancer burden was estimated to have risen to 20 million new cases of cancer in 2022, suggesting that 1 in 5 individuals develop cancer in a lifetime [1]. Concurrently, advances in oncological therapies and early detection have led to a marked reduction in mortality rates, with an estimated 69.9% surviving at least 5 years after diagnosis [2]. As survivorship increases, the goals of oncology care are expanding beyond prolongation of life to encompass the holistic well-being of patients [3]. Particularly, cancer and its treatments are frequently associated with a broad spectrum of psychosocial stressors, including but not limited to loss of autonomy, heightened anxiety, depression, and disruptions in social relationships, which can profoundly influence disease trajectories, treatment adherence, and quality of life [4]. The pressure to achieve expected life transitions at specific stages can intensify persistent feelings of frustration and social isolation, especially when illness prevents them from making certain progress [5]. Against this backdrop, it is essential to systematically characterize how patients experience and articulate these psychosocial issues for developing responsive, psycho-oncology services and targeted supportive interventions.

Traditional research on psychosocial issues in oncology has relied heavily on interviews and surveys. While these methods offer in-depth and clinical validity, they are inherently constrained by their scope, scalability, and susceptibility to recall bias. These limitations compromise the ability to capture nuanced, evolving experiences among diverse populations, particularly for sensitive or underreported topics such as sexual health concerns, fertility fears, financial toxicity, and intimate partner dynamics. With the global growth of internet use, many patients with cancer now share their cancer experiences in online health communities (OHCs) to seek information, empathy, and peer support. These patient-authored narratives often reveal emergent concerns and unmet needs, offering a unique lens on lived experience, values, and priorities with a bottom-up approach that traditional data sources may fail to access. However, the resulting qualitative corpora are typically large-scale, heterogeneous in style and content, and characterized by irregular structure and variable quality, where traditional qualitative analytics may struggle with subjectivity, timeliness, and reproducibility when applied at scale.

Recent advancements in natural language processing, including topic modeling and large language models (LLMs), enable scalable analysis of unstructured text to identify latent themes, sentiments, and temporal dynamics. Unlike conventional topic models (eg, latent Dirichlet allocation [LDA]), which represent topics as unordered collections of words and offer limited user control over topic granularity and interpretability, TopicGPT uses a prompt-based, LLM-driven framework generating topic hierarchies that are more coherent, clinically meaningful, and better aligned with human categorizations [6-8]. By incorporating specific lexicons as well as domain-adapted prompts, it has demonstrated superior performance in extracting nuanced, interpretable themes in specific domains. For example, Shokri et al [6] used TopicGPT with modified prompts and an initial set of topics to identify common patterns in domestic violence narratives, resulting in 83 different topics, such as psychological aggression, financial abuse, and physical assault, from 1576 patient stories. Pham et al [7] demonstrated that TopicGPT outperformed 3 popular topic models: LDA, SeededLDA, and BERTopic in generating topics from 2200 sampled Wikipedia articles and bill summaries, achieving the highest alignment with ground-truth labels across all metrics. Building on its strengths, TopicGPT offers a novel and efficient avenue to analyze large-scale patient narratives while maintaining contextual sensitivity. However, its applications in health care, particularly within the field of psycho-oncology, remain largely unexplored, representing a critical gap in the literature.

To address this gap, this study proposes an integrated, domain-adapted pipeline that combines TopicGPT with network analysis to examine psychosocial issues encountered by patients with cancer. Leveraging state-of-the-art LLMs, this study aims to use TopicGPT to generate coherent, clinically interpretable themes from patient-authored narratives and map them at the sentence level to identify major psychosocial challenges discussed in relation to cancer types with high global prevalence. Furthermore, the study will construct a network, in which the resulting topics serve as nodes, and their co-occurrences form the edges, enabling the modeling of topic relationships. The findings from this research have the potential to advance computational analysis of large-scale qualitative data in health care and provide data-driven insights through intuitive visualizations for clinicians, patients, and policymakers.

## Methods

### Study Design

**Figure 1.**
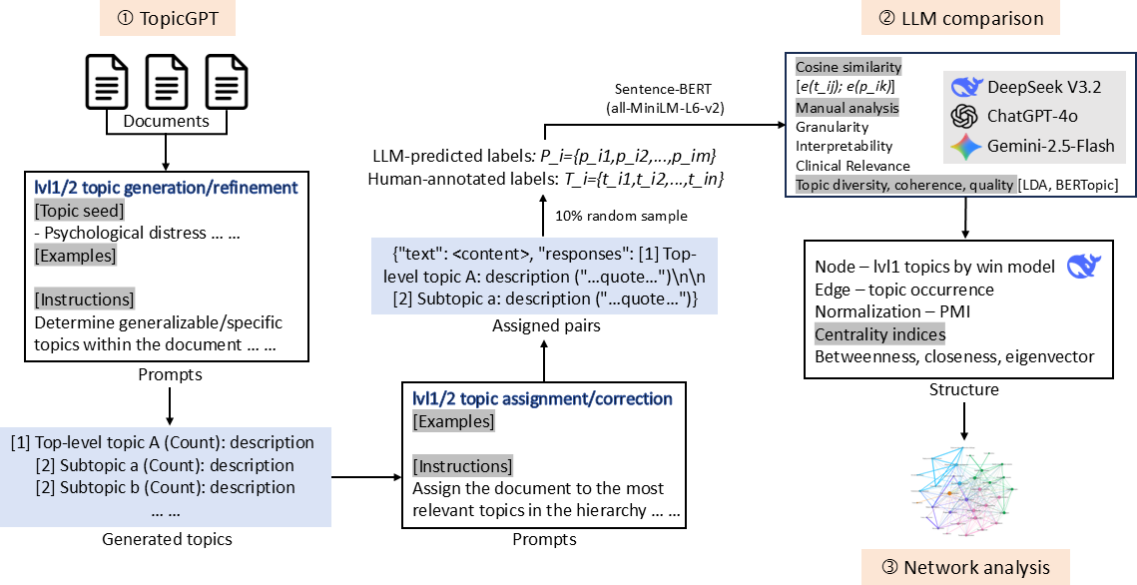
The methodology workflow. LLM: large language model.

This is a retrospective study using publicly available, user-generated narratives from OHCs to identify psychosocial themes in cancer experiences and model their interrelationships. Topic generation, assignment, and categorization were performed using a modified version of TopicGPT. To identify the best-performing LLM, we compared their performance by computing cosine similarity scores between LLM-predicted labels and human-annotated labels, followed by a manual evaluation. Topic co-occurrence network analysis was conducted subsequently to elucidate the influential topics and their interrelationships across patient narratives. Figure 1 presents the entire methodology workflow.

### Data Collection and Preprocessing

Data were collected from publicly available OHCs (eg, Reddit, Macmillan Cancer Support, Mijian, and Douban) that permit research use in accordance with ethical guidelines. The posts were crawled on September 26, 2025, following institutional review board approval. As this is a retrospective study, accessible posts published prior to the date of data collection were included. We focused on discussion spaces dedicated to breast cancer, cervical cancer, and thyroid cancer. Relevant posts were identified either through preexisting disease-specific subforums on each platform or through platform-specific keyword searches using combinations of terms such as “breast,” “cervix,” “cervical,” “thyroid,” “cancer,” “tumor,” and “carcinoma” as well as their Chinese equivalents where applicable. For each post, the username, posting date, title, main content, comments, and URLs were extracted using Python (version 3.12; Python Software Foundation). To ensure privacy, any personally identifiable information including names, contact information, addresses, and workplaces in the dataset were removed, and all usernames were replaced with randomly generated user IDs. The process was configured to handle bilingual datasets without loss of information.

Collected posts were manually screened by 6 independent research assistants who received standardized training based on a shared coding protocol. Relevant characteristics were systematically documented in a structured spreadsheet, including demographic information (eg, age and gender), disease trajectory (eg, diagnosis, treatment, remission, recurrence, and end-of-life care), treatment-related information (eg, surgery, chemotherapy, radiotherapy, and hormone therapy), where available, and whether psychosocial concerns were present (yes or no). Inclusion criteria were as follows: (1) posts describing psychosocial issues related to cancer diagnosis, treatment, remission, recurrence, or end-of-life care; (2) posts written in English or Chinese; and (3) posts authored by individuals diagnosed with cancer. To determine whether a post was patient-authored, screeners relied on explicit self-identification and first-person descriptions of personal cancer experiences (eg, “I was diagnosed” and “my side effects”). Exclusion criteria include (1) posts containing fewer than 10 words or those lacking sufficient content to analyze psychosocial issues; (2) posts authored by family members of patients with cancer or health care professionals; (3) advertisements, product promotions, reports of cell or animal experiments, clinical trial or survey recruitment, and purely informational posts; (4) help-seeking posts without articulating any psychosocial issues; (5) posts describing cancer experiences in nonhuman subjects (eg, animals); (6) posts not relevant to cancer; and (7) duplicates, identified as posts containing identical text from the same author on the same platform. These records were manually reviewed and removed to avoid redundant inclusion. Throughout the screening process, the authors (XF and VH) held biweekly meetings with the screeners to discuss uncertain cases and to check for potential screening errors.

### TopicGPT

Eligible patient-authored posts were converted into JSONL format, with emojis, URLs, commercial at symbols, and extra whitespace removed to standardize the documents. These cleaned documents were then passed to 3 different state-of-the-art LLMs (ie, DeepSeek-V3.2, GPT-4o, and Gemini-2.5-Flash) for topic modeling using *topicgpt_python* package. We used a slightly modified version of TopicGPT to excavate topics from the patient narratives, and models were run with the default generation settings provided by each application programming interface [7] (Multimedia Appendix 1). Instruction prompts were carefully tailored to align with our research objectives at each step, and a seed file containing a predefined small set of initial topics was provided to guide the models and ensure the relevance of generated topics [7]. To enhance interpretability and minimize overlaps among topics, the refinement phase was divided into 2 separate tasks for both top-level topics and subtopics, resulting in a six-step process:

1. Top-level topic generation: models were prompted to generate broad, generalizable topics that can accommodate further subtopics, each followed by a brief description. For example, “[1] Psychological distress: Mentions a set of painful mental and physical symptoms that are associated with normal fluctuations of mood in most people,” with definitions sourced from the American Psychological Association dictionary of psychology.
2. Top-level topic refinement: generated topics were refined by merging paraphrased or duplicate topics into a new or existing one and removing irrelevant topics.
3. Subtopic generation: if existed, given the refined level-1 list, more specific subtopics were generated under high-level topics with brief descriptions. This step aims to add actionable granularity, where analysis, interventions, and resource mapping happen at this level. For example: “[1] Psychological distress [2] Anxiety (Document: 1, 3): Mentions an emotion characterized by apprehension and somatic symptoms of tension in which an individual anticipates impending danger, catastrophe, or misfortune.” If a top-level topic was already specific enough or duplicated, no subtopics were added.
4. Subtopic refinement: subtopics were further refined in a similar manner, with new topics created only if no existing topics applicable to a post. It aims at cleaning long tails and collapsing near-duplicates since level-2 is where duplication explodes (“fear of recurrence,” “cancer coming back,” and “recurrence worry”). This refinement setting could improve efficiency and prevent cross-parent merges (ie, serious errors of merging topics from different levels).
5. Topic assignment: topics at each level were counted individually for identifying the most frequently discussed themes. Refined topics were assigned to the corresponding input text, supported by a quoted sentence as rationale. For example: “[1] Fear of cancer recurrence: Mentions the fear or worry that cancer could return or progress in the same place or another part of the body. (‘... the thought of reoccurrence would eat me alive everyday and not let me live.’” No new topics were allowed in this step.
6. Topic correction: finally, models were reprompted to review and correct topic-quote pairs, ensuring that assignments were consistent with the established topic hierarchy.

### LLM Performance Comparison

To evaluate LLM performance in topic modeling, we compared the outputs of different LLMs with a reference set independently annotated by 2 researchers (XF and CKC) with 8-year expertise in health care and psychological counseling, who received standardized training and used a predefined codebook as a reference. The codebook was developed and refined through repeated discussion among the research team, while the coders were allowed to remain open to new concepts emerging from the data. Interrater agreement was calculated using macroaverage Cohen κ [9]. Semantic similarity between the predicted topic clusters and the human-annotated labels was assessed using the cosine similarity metric [10]. To this end, 10% of the dataset was randomly selected through automated random number generation. For each document *i* in the selected dataset, we obtained 2 sets of topic labels: the LLM-predicted labels $P_{i}=\{\;p_{i1},\;p_{i2},\;\ldots ,\;p_{is}\;\}$ and human-annotated labels $T_{i}=\{\;t_{i1},\;t_{i2},\;\ldots ,\;t_{is}\;\}$. To capture the semantic meaning of each label, we encoded each label into a dense vector representation using Sentence-Bidirectional Encoder Representations from Transformers (BERT) “all-MiniLM-L6-v2” [11]. Specifically, each human label and each LLM label was transformed into embeddings $e\;\left(t_{ij}\right)$ and $e\;\left(p_{ik}\right)$, respectively. To represent the overall semantic content of the human and LLM label clusters for each document, we aggregated the embeddings within each set by computing the mean vector:


(1) $$E_{T_{i}}=\frac{1}{n}\sum_{j=1}^{n}e\;\left(t_{ij}\right)$$

(2) $$E_{P_{i}}=\frac{1}{m}\sum_{k=1}^{m}e(p_{ik})$$

where *n* and *m* denote the number of human and LLM labels for document *i*, respectively. The semantic similarity between the human and LLM label clusters for each document was then quantified using cosine similarity, defined as:


(3) $${\text{Cosine similarity}}_{i}=\frac{E_{T_{i}}\cdot E_{P_{i}}}{\left\|E_{T_{i}}\|\|E_{P_{i}}\right\|}$$

where $E_{T_{i}}\cdot E_{P_{i}}$ represents the dot product of 2 mean embedding vectors; $\left\|E_{T_{i}}\right\|$ and $\left\|E_{P_{i}}\right\|$ denote their Euclidean norms. The potential similarity ranges from −1 to 1, with values closer to 1 indicating that the vectors are more aligned in the same direction. To obtain an overall measure of semantic alignment, we calculated the average cosine similarity across all sampled documents (*i*=1, 2, ..., *N*). Both top-level topics and subtopics were evaluated to comprehensively assess the LLM’s ability to generate nuanced and fine-grained topics in comparison to human annotators. All embeddings were generated using the sentence-transformers Python library with the “all-MiniLM-L6-v2” model.

To further elucidate the tendencies of distinct models in topic identification, we also conducted a manual analysis of the generated topics to provide additional insights into their clinical relevance, granularity, and interpretability. In this study, clinical relevance refers to how relevant and actionable the information is for clinical care. Granularity refers to the size and the level of detail captured by the topics. Interpretability refers to how well humans can make sense of the topics generated by the model. For each attribute, the models were independently ranked by 2 researchers (XF and HCK), and interrater agreement was assessed using the Spearman rank correlation coefficient.

### Baseline Comparisons With LDA and BERTopic

Two widely used topic modeling approaches were used as baselines: the probabilistic LDA [12] and the transformer-based BERTopic [13]. Given that the dataset contained bilingual posts, both models were applied separately to each language subset. To enable a fair comparison, we followed the approach of Pham et al [7] by setting the number of topics *k* equal to the number of topics generated by the best-performing TopicGPT for each language. Raw text was preprocessed using the same pipeline for both models, which included lowercasing, tokenization, removal of a custom stop-word list, and lemmatization. A grid search was conducted over the Dirichlet hyperparameters α (document-topic prior) and η (topic-word prior). The candidate sets were α∈{0.10, 0.12, 0.14, 0.16, 0.18, “auto”} and η∈{0.10, 0.12, 0.14, 0.16, 0.18, “auto”}. For each combination, an LDA model was trained using 10 passes and 50 iterations. Model performance was evaluated using the *c_v_* topic coherence score [14]. The hyperparameter pair that yielded the highest coherence was selected as optimal. A final LDA model was then trained with the optimal hyperparameters using passes=20, iterations=100, and a random state of 42 for reproducibility. Interactive visualizations were generated with the pyLDAvis library to inspect topic distances and term relevance. For BERTopic, document embeddings were produced using either the English-only “all-MiniLM-L6-v2” or the multilingual “paraphrase-multilingual-MiniLM-L12-v2” [11], which output 384-dimensional sentence vectors. Dimensionality reductions were performed with Uniform Manifold Approximation and Projection, and the reduced embeddings were then clustered using K-Means to language-specific *k*. A 2D Uniform Manifold Approximation and Projection scatter plot was generated to visualize the document embeddings, with each point colored by its assigned topic.

For both models, we computed the *c_v_* coherence score with CoherenceModel in the Gensim library. For each topic, the top 15 words were extracted. The measure calculates the normalized pointwise mutual information (PMI) for all word pairs within the corpus, and the overall model coherence is then reported as the arithmetic mean of the coherence scores of all topics [14]. Because TopicGPT outputs natural language topic labels rather than a ranked list of keywords derived from a learned vocabulary distribution, the coherence metric is not applicable to it. Instead, the topic diversity measure based on semantic embedding was adopted to enable fair comparison across all 3 methods. Each topic was represented as either a concatenation of its top 15 words (LDA and BERTopic) or as its natural language label (TopicGPT). Topic representations were encoded with “all-MiniLM-L6-v2” or “paraphrase-multilingual-MiniLM-L12-v2” [11], and the diversity was computed as [15]:


(4) $$\text{Topic diversity}=1-\frac{2}{k(k-1)}\sum_{i§lt;j}\cos(v_{i},v_{j})$$

where *k* is the total number of topics, $v_{i}$ denotes the embedding vector of the *i*th topic, and $cos(v_{i},v_{j})$ is the cosine similarity between 2 topic vectors. A value close to 1 indicates high semantic distinctness, whereas a value near 0 suggests that most topic vectors are similar, implying that the model produces highly overlapping or duplicate themes. Finally, the topic quality score was computed by the topic coherence and topic diversity [16].

### Topic Co-Occurrence Network Analysis

Following the finalization of topic assignment, topic co-occurrence network analysis was conducted to examine the structural relationships among topics emerging in our dataset. Since a single sentence can generate both top-level and more specific subtopics within the topic hierarchy, we included only the broader top-level topics when building the network to minimize the impact of overlapping quotes on topic interrelationships. In this network, nodes represent the distinct top-level topics identified through TopicGPT, while edges represent the topic of co-occurrence within the same documents. PMI was used to normalize the edge weights and quantify the statistical dependence between 2 topics by comparing their observed co-occurrence probability against the expected probability under the assumption of independence [17]. This approach goes beyond simple co-occurrence counts by distinguishing meaningful thematic relationships from random, coincidental correlations that may result from high-frequency topics. The PMI is calculated as:


(5) $$PMI(x;y)=\log_{2}\frac{p(x,y)}{p(x)p(y)}$$

In this formula, *p(x,y*) is the probability that topics *x* and *y* co-occur, *p(x*) and *p(y*) refer to the probability of occurrence of topic *x* and *y*, respectively. Edges with positive PMI values (PMI>0) were retained, corresponding to topic pairs that co-occur more frequently than expected by chance. By transforming the edge weights from raw frequency of co-occurrence to PMI scores, we constructed a network where connections represent semantically meaningful relationships [6,18]. The PMI values were calculated and visualized using Python (version 3.12).

Multiple centrality indices were calculated to evaluate the structure of psychosocial networks and identify the most influential topics derived from the narratives of patients with cancer. Specifically, degree centrality counted the number of direct connections each node has within the network, identifying the most frequently co-occurring issues [19]. The weighted degree centrality, also node strength, was calculated as the sum of transformed edge weights to identify topics that most frequently, nonrandomly co-occur with other topics across the dataset [20]. To evaluate the overall connectivity of the network, network density was reported, which is defined as the ratio of the number of observed edges to the maximum possible number of edges in a fully connected network [21]. Closeness centrality was calculated as the reciprocal of the sum of the length of the shortest paths between the node and all other nodes. The closer a node is to all other nodes, the more central it is within the network [22]. Betweenness centrality measured how frequently a node appears on the shortest path between other nodes in the network. A node with higher betweenness centrality would have more control over the network, representing a critical point of intersection or transition in patient experience [23]. Eigenvector centrality was calculated to identify influential topics that are connected to other well-connected topics, where higher values indicate that the node is connected to many nodes that themselves have high scores [24]. The network visualization and centrality calculation were achieved using Gephi software (version 0.10.1) [25].

### Ethical Considerations

This study was granted ethics approval by the Hong Kong Polytechnic University Institutional Review Board (HSEARS20250904002). The study exclusively used publicly accessible data from OHCs that explicitly permit research use in accordance with their terms of service. Therefore, individual informed consent was not feasible and was waived. Any personally identifiable information, including usernames, locations, or references to third parties, was removed from the dataset. All study data were stored in password-protected files accessible only to the research team.

## Results

### Overview

A total of 11,306 posts were collected from publicly accessible online platforms, including Reddit, Macmillan Cancer Support, Mijian, and Douban, spanning the period from December 6, 2006, to September 24, 2025. We focused our data collection on subforums related to breast cancer (n=4172), cervical cancer (n=3827), and thyroid cancer (n=3307), given their high global prevalence and the substantial volume of user discussions within OHCs. A rigorous screening process was conducted based on eligible criteria to ensure relevance to psychosocial stressors described by patients themselves, resulting in 3169 posts being retained for topic modeling.

### LLM Performance Comparison

#### Semantic Similarity

Three state-of-the-art LLMs were used to generate and classify topics and subtopics. Table 1 summarizes the results obtained from DeepSeek-V3.2, GPT-4o, and Gemini-2.5-Flash. Specifically, DeepSeek-V3.2 demonstrated the highest capacity for identifying unique topics, with 42 top-level topics (6559 total occurrences) and 58 subtopics (7326 total occurrences). GPT-4o produced the lowest number and occurrences of top-level topics while yielding the highest number of overall subtopic occurrences. Macroaverage Cohen κ was 0.462 (95% CI 0.440‐0.546). Most disagreements reflected differences in label use, coding granularity, or interpretation of nuanced patient narratives; these cases were resolved through discussion with a faculty member to achieve consensus. Semantic alignment between LLM-predicted labels and human-annotated labels was calculated across 3 models using cosine similarity over Sentence-BERT embeddings. Among them, DeepSeek-V3.2 achieved the highest alignment (0.6295), followed by Gemini-2.5-Flash (0.5342).

**Table 1.** TopicGPT performance based on 3 state-of-the-art large language models.

Metrics	DeepSeek-V3.2	GPT-4o	Gemini-2.5-Flash
Top-level topics (occurrences)	42 (6559)	12 (1205)	17 (4986)
Subtopics (occurrences)	58 (7326)	21 (7972)	28 (5347)
Average cosine similarity	0.6295	0.5247	0.5342

To better understand the causes of misalignments, we traced a few samples with low similarity. First, misunderstandings mostly arise from overinterpretation or distortion of patient intent. For example, for “I’ve been told I will be having a total thyroidectomy with bilateral neck dissection and was just curious if anyone could tell me what kind of scarring would be left with” (Post #1877), the models assigned the topics “Fear of medical procedures,” “Recovery concerns,” and “Emotional and psychological impact.” Second, the models occasionally skipped over the direct intent for less relevant cues. For example, for “Anyone try out fake nipples? ... I do feel a little alien not having them *...* If you wore silicone (or other material) nipples where did you buy them? And what did they feel like going about daily life?” (Post #2271), the models assigned labels such as “Quality of life impact” and “Physical symptoms,” but failed to capture the primary intent to seek advice and practical support. Another example is “Why I’m in my head, is my doctor said it was my choice to do it or not which I thought was weird *...*. When I asked what if I have cancer and wait, she said well it might grow and it’s too big for RAI” (Post #1318); the models assigned the labels “Fear of cancer recurrence” and “Emotional and psychological impact,” which failed to address the core issue of uncertainty or confusion regarding decision-making. Nevertheless, some misalignments are to be expected, given a single sentence can be interpreted in multiple ways. For instance, for “Can anyone give me some idea how to get through chemo radiation. Are you given help with nausea etc.” (Post #781), the human annotator labeled this as “Information seeking,” while the model assigned the label “Treatment side effects,” which is also semantically relevant.

#### Granularity, Interpretability, and Clinical Relevance

Interrater agreement on the rankings was perfect for granularity and interpretability (Spearman ρ=1.0) and moderate for clinical relevance (Spearman ρ=0.5). DeepSeek-V3.2 was identified as contributing the most interpretable, fine-grained, and clinically relevant topics, while the other 2 models demonstrated distinct tendencies in interpreting patient experiences, often exhibiting limited scope and less actionable insights. Table 2 shows examples of post excerpts alongside their corresponding topics, as generated by the models and human annotators. Particularly, Gemini-2.5-Flash excelled in providing detailed breakdowns of physical symptoms (eg, “Hairloss,” “Vomiting,” “Nausea,” “Numbness,” and “Bleeding”) and generated mutually exclusive subtopics more effectively than the other models. However, this often resulted in overly fragmented clusters, making it hard to derive holistic insights. Additionally, Gemini-2.5-Flash struggled to address sensitive and contextual observations, such as issues related to support-seeking behaviors, decision-making conflicts, and interactions between patients and the health care environment (Posts #2506, #2882, and #2470; Table 2). GPT-4o, while assigning the highest frequency of subtopics across documents, tended to produce topics that were overly concise or excessively broad. This lack of granularity limited the results in reflecting the specific issues experienced by patients. For instance, subtopics such as “Emotional and psychological impact,” “Treatment and side effects,” “Diagnosis and staging,” and “Medical dismissal and validation” were too generalized (Posts #749 and #2587; Table 2), leading to a loss of practical and interpretable insights. In contrast, DeepSeek-V3.2 consistently outperformed the others in generating the most informative, contextual, and actionable perspectives. For example, it identified topics such as “Patient-provider communication challenges” and “Social communication and support” under the broader category of “Health care communication” (Post #2506; Table 2), offering a clear roadmap for addressing communication gaps in both clinical and social settings. Similarly, in Post #749, it captured multiple layers of patient experiences within a single narrative, including “Disclosure anxiety,” “Job retention challenges,” and “Diagnostic delays and misdiagnosis.” This level of detail delivers a comprehensive understanding of patient journeys across medical, emotional, and social dimensions, which is critical for designing effective support systems for individuals undergoing treatment.

**Table 2.** Examples of topics generated by large language models and human annotators.

Post excerpts	DeepSeek-V3.2	GPT-4o	Gemini-2.5-Flash	Human
“When I called for the results, I was reassured that I had fibroids and was asked if I wanted a gynea referral! *...* I lost of job as I wasn’t able to do it with the pain *...* I was diagnosed with stage 3b cervical cancer *...* I called my surgery and a very kind doctor read the report that came with my initial scan results. ‘Mass detected, possible fibroids’. You can imagine my anger *...* I haven’t told the children about the cancer *...* I can’t tell their father. I’m scared he’ll try to take the children from me *...* I can’t bear the thought of not seeing my children grow up .... I feel I’m at breaking point” [Post #749].	Patient-provider communication challenges; disclosure anxiety; job retention challenges; diagnostic delays and misdiagnosis; fear of cancer recurrence	Medical dismissal and validation	Emotional impact; strained relationships; reproductive concerns	Psychological distress; family dynamics
“I can’t go to work as the chemotherapy makes me unwell for a week or two, I’ve heard the symptoms can get worse the longer the process! *...* Could I get refused because I’m only sick for a couple of weeks but things may change example lethargic or other symptoms. I really can’t understand how someone going through cancer treatment should be refused PIP but I’ve heard so many do. I am on full pay from work until July and my worry is when my income goes to half pay I’m going to struggle financially” [Post #2470].	Financial strain; fatigue and weakness; employment concerns; treatment side effects	Treatment and side effects; emotional and psychological impact	Physical symptoms; fatigue	Anxiety; information seeking
“I can’t stand when people say ‘you’ve got this’ when I don’t or my aut response is ‘but I don’t want cancer!’ Or when people make decisions/assume how I’m feeling without asking me!” [Post #2506].	Health care communication; patient-provider communication challenges; social communication and support	Communication challenges	—^a^	Frustration with unhelpful encouragement; lack of decision-making autonomy
“I have found the change in routine and silence really hard. There’re no appointments apart from my surgery in the diary” [Post #2587].	Treatment isolation	Emotional and psychological impact	Psychological distress	Posttreatment adjustment issues
“Should I have been warned about possible pain? The whole thing felt unprofessional *...* burst into tears from physical shock ... waking up in the middle of the night anxious about it all” [Post #2882].	Patient-provider communication challenges; sleep disturbance; psychological distress	Medical dismissal and validation; anxiety	Psychological distress; anxiety; sleep issues	Unprofessional health care; insufficiently informed; psychological distress

a Not available.

In this study, TopicGPT demonstrated significant advantages over manual annotations in generating more comprehensive, consistent, and structured topics from patient narratives. Human annotations excelled in capturing underlying emotions and intentions, such as “Lack of decision-making autonomy” and “Frustration with unhelpful encouragement” (Post #2506; Table 2). However, human annotations occasionally missed critical information and often failed to provide consistent outputs. For example, in Posts #749 and #2470, human annotations missed critical aspects in concerns about employment, disclosure, and financial burden, which, however, were successfully identified by DeepSeek-V3.2. While human annotations sometimes provided unique and deep clinical insights, their inability to produce comprehensive and structured outputs limited their overall utility in this context. Based on such findings, the results from DeepSeek-V3.2 were chosen to construct the psychosocial network. Figure S1 in Multimedia Appendix 2 presents the top 20 most frequent top-level topics in patient narratives generated by DeepSeek-V3.2. Prevalent topics include “Fear of cancer recurrence,” “Psychological distress,” “Treatment decision concerns,” “Diagnostic concerns,” and “Treatment side effects.” The specific frequencies of the top 20 topics are presented in Table S2 in Multimedia Appendix 2. A subgroup analysis by language was performed for English and Chinese posts. In both groups, “Fear of cancer recurrence” and “Psychological distress” were among the most common topics. Other frequent topics included “Treatment decision concerns,” “Diagnostic concerns,” and “Treatment side effects” in English posts, and “Quality of life impact,” “Social isolation,” and “Coping strategies” in Chinese posts.

### Baseline Comparisons With LDA and BERTopic

Table 3 summarizes the quantitative comparison between TopicGPT (DeepSeek-V3.2) and the 2 baseline models for the English and Chinese subsets. For the English corpus (k=42), BERTopic showed slightly higher topic coherence (0.594 vs 0.437) and overall topic quality (0.451 vs 0.351) than LDA. Although LDA obtained marginally higher topic diversity than TopicGPT (0.804 vs 0.788) on the English corpus, this difference is small and likely reflects differences in topic representation rather than a true loss of thematic breadth. For the Chinese corpus (k=21), both baseline models showed reduced topic diversity than in English. BERTopic again outperformed LDA in topic coherence (0.513 vs 0.403) and overall topic quality (0.272 vs 0.213). TopicGPT maintained consistently high diversity across languages, with the highest score of 0.759, indicating that its labels capture a broad and nonredundant set of themes that are comparable to or exceeding the baseline models. Interactive visualizations for 2 baseline models are shown in Figures S1-S4 in Multimedia Appendix 3. Table S1 in Multimedia Appendix 2 provides example topic assignments from the 3 models, illustrating that both baseline methods ultimately represent topics as ranked lists of salient keywords derived from term statistics within the corpus, whereas TopicGPT directly produces interpretable natural language labels for each topic.

**Table 3.** Comparison of model performance.

Dataset and models	Mean pairwise cosine (SD)	Topic diversity	Topic coherence	Topic quality
English (n=2936; k=42)				
TopicGPT	0.212 (0.147)	0.788	N/A^a^	N/A
LDA^b^	0.196 (0.118)	0.804	0.437	0.351
BERTopic	0.241 (0.153)	0.759	0.594	0.451
Chinese (n=232; k=21)				
TopicGPT	0.241 (0.140)	0.759	N/A	N/A
LDA	0.472 (0.174)	0.528	0.403	0.213
BERTopic	0.469 (0.172)	0.530	0.513	0.272

a N/A: not applicable.

b LDA: latent Dirichlet allocation.

### Topic Co-Occurrence Network Analysis

**Figure 2.**
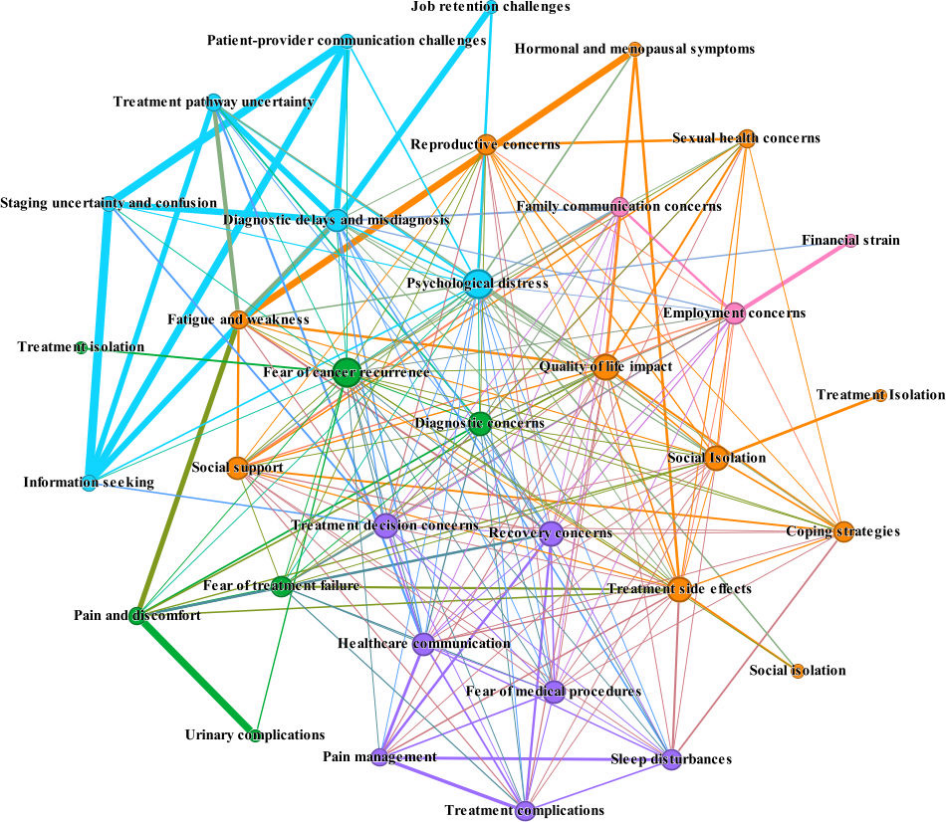
The topic network structure (DeepSeek-V3.2).

The overall network structure laid out by the Fruchterman-Reingold algorithm is presented in Figure 2. The size of the node denotes the degree centrality, with degrees ranging from 1 to 30. The network density was 0.406 after filtering the node with a degree of 0, indicating a moderate level of connectivity where approximately 40.6% of all possible connections among nodes were present. Table S2 in Multimedia Appendix 2 lists the top 20 influential topics based on multiple centrality indices. Across the various metrics, concerns related to medical procedures, side effects and complications, employment, disease progress, social support, and communication majorly constituted the influential group, followed by psychological distress, reproductive and sexual health, and decision conflicts.

**Figure 3.**
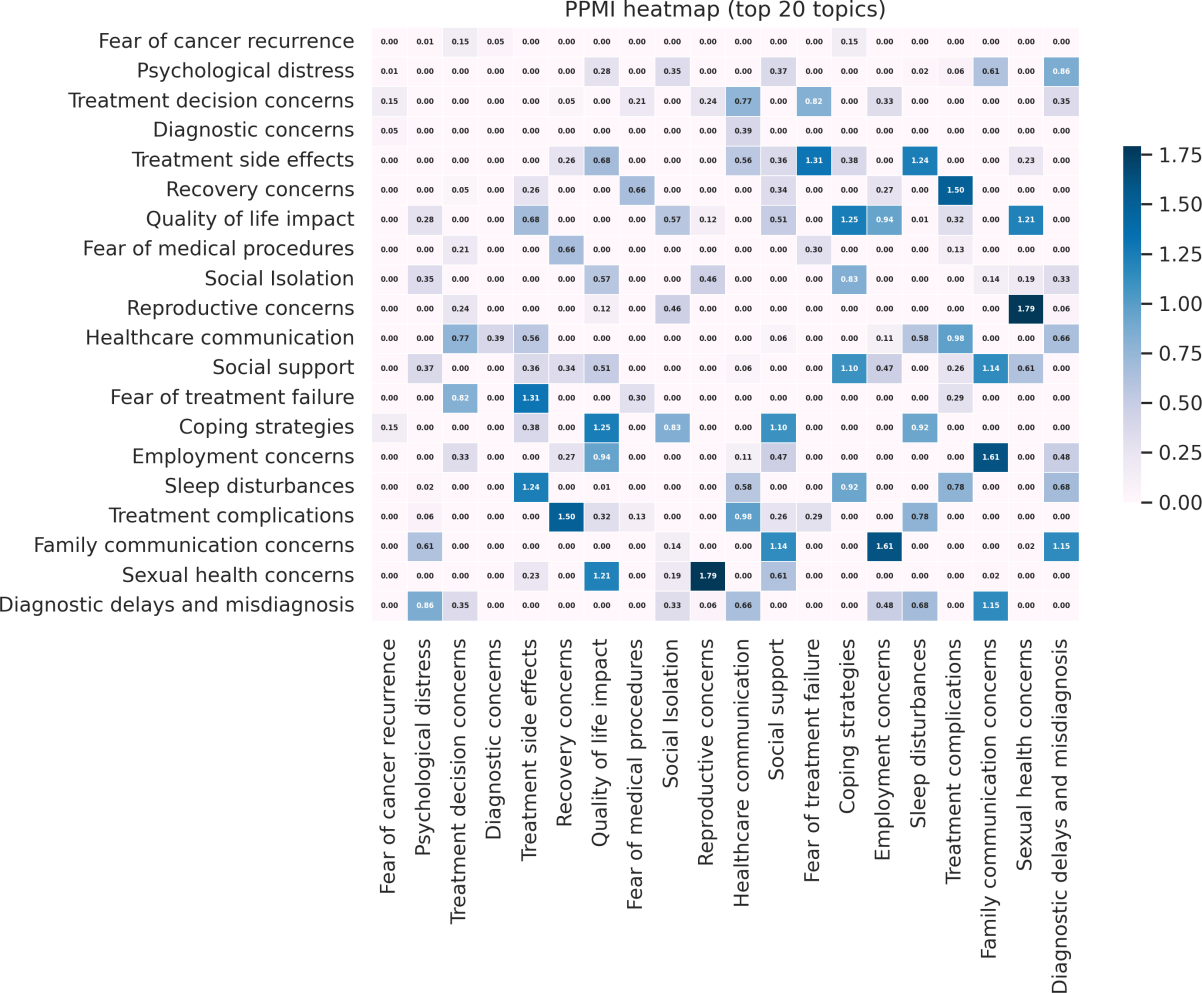
The PPMI heatmap of topic relationships (DeepSeek-V3.2). PPMI: positive pointwise mutual information.

Figure 3 presents the positive PMI heatmap, illustrating the relationships among the top 20 most frequent topics in patient-authored posts. Darker shades represent stronger-than-expected associations between topics. Specifically, “Sexual health concerns” frequently co-occurred with both “Reproductive concerns” (PMI=1.79) and “Quality of life impact” (PMI=1.21). Similarly, “Family communication concerns” often appeared alongside “Employment concerns” (PMI=1.61), “Diagnostic delays and misdiagnosis” (PMI=1.15), and “Social support” (PMI=1.14). “Coping strategies” were commonly discussed together with “Quality of life impact” (PMI=1.25) and “Social support” (PMI=1.10). “Treatment side effects” frequently co-occurred with “Sleep disturbance” (PMI=1.24) and “Fear of treatment failure” (PMI=1.31). In addition, strong connections were observed between “Recovery concerns” and “Treatment complications” (PMI=1.50). Interestingly, although some topics, such as “Fear of cancer recurrence,” “Psychological distress,” and “Treatment decision concerns,” exhibit a strong weighted degree, they demonstrate very low PMI values. The extensive connections observed among these topics are primarily due to their high frequency within the corpus, rather than genuine semantic relationships with other topics.

## Discussion

### Principal Findings

This study leveraged a modified version of TopicGPT, an innovative LLM-based topic modeling framework, to derive topics from large-scale, unstructured qualitative health care data. By contextually understanding and linking each meaningful sentence within documents to a specific topic, TopicGPT allows researchers to trace the reasoning behind topic assignments, enhancing the interpretability and transparency of topic modeling. From a technical perspective, it overcomes the limited input window inherent to standard LLMs, enabling the processing of lengthy and sizable health care narratives without sacrificing contextual richness. More importantly, it offers substantial efficiency gains over manual qualitative coding, which is often labor-intensive, time-consuming, and subject to interannotator variability. By automating the extraction of sentence quotes and assigning a refined topic, TopicGPT not only accelerates the analytic process but also ensures greater consistency and scalability, providing an effective, reproducible framework for health care qualitative analysis.

To identify the most effective topic hierarchy, we compared the performance of 3 state-of-the-art LLMs, DeepSeek-V3.2, GPT-4o, and Gemini-2.5-Flash, by quantitatively measuring the similarity between model-predicted topic classes and human-annotated labels, as well as qualitatively analyzing their respective tendencies in topic identification. This comparative evaluation revealed marked differences in the models’ abilities to interpret the complexity of patient experiences and generate granular, clinically relevant topics. Notably, Gemini-2.5-Flash demonstrated proficiency in pinpointing specific physical symptoms, while GPT-4o excelled at extracting salient narrative segments. However, both models exhibited limitations in their coverage of the broader psychosocial context and often lacked the depth necessary for actionable clinical insights. A critical observation was that the topic hierarchies produced by these models were heavily influenced by the initial set of topic seeds. This sensitivity likely stems from our deliberate decision to supply only a minimal set of topic seeds, following the approach suggested by Pham et al [7], to balance generalization and specificity without restricting the models to a particular domain. As a result, their outputs were narrower in scope and off track than anticipated. In contrast, DeepSeek-V3.2 consistently outperformed the other models within our evaluation framework, providing the most informative and contextually nuanced topic categorizations, which reflected a heightened sensitivity to the layered and interconnected nature of patients’ lived experience. This capability makes DeepSeek-V3.2 particularly well-suited for exploring the intricate relationships among the psychosocial challenges encountered by patients with cancer. Ultimately, our TopicGPT experiments carry significant implications for the application of LLMs in clinical narrative analysis. Current LLMs take substantial advantages over human annotations especially in terms of efficiency, consistency, and generation of structured insights, presenting considerable promise for automating the extraction of insights from patient narratives. Nevertheless, their effectiveness is contingent upon both model architecture and prompt engineering. The observed model-specific tendencies underscore the necessity of careful selection and configuration in accordance with the desired analytical depth and domain coverage. For example, summarizing highly specific details, such as symptoms, severity, and subtypes, from clinical documentation may require models optimized for granularity, whereas thematic analysis of unstructured patient narratives or stakeholder perspectives may benefit from models capable of producing more holistic yet actionable insights.

Moreover, we compared the outputs of the best-performing TopicGPT with those of LDA and BERTopic. Overall, TopicGPT demonstrates the ability to consistently produce interpretable and semantically distinct topics. The observed differences in topic diversity between TopicGPT and the baselines should be interpreted in light of how topics are represented and embedded for evaluation. Notably, the diversity metric is computed on embedding-based similarities between topic text representations. For LDA and BERTopic, each topic is expressed as a list of high-probability keywords, which we concatenate into a long text string before embedding, yielding representations that capture a broad mixture of lexical content. Additionally, LDA topics often blend multiple dimensions within a single keyword list (eg, “topic_9”: [“people,” “cancer,” “day,” “time,” “friend,” “life,” “say,” “support,” “family,” “child,” “diagnosis,” “work,” “hard,” “today,” and “treatment”]). This lexical heterogeneity further increases intertopic distances in the embedding space and can inflate the diversity. By contrast, TopicGPT represents each topic with a concise natural language label, which is highly compressed and thematically focused. As a result, TopicGPT may yield slightly lower diversity values, even though at the document level it can assign multiple complementary labels that more fully capture the multifaceted nature of individuals’ concerns.

To further explore how emerging psychosocial factors interrelate in patient narratives, we constructed a topic co-occurrence network based on outputs from the best-performing LLM (ie, DeepSeek-V3.2) to identify influential topics through multiple centrality measures, PMI values, and network visualization. Overall, our findings are broadly consistent with prior research in demonstrating that psychological distress, symptom burden, and informational needs remain central features of online cancer discourse [26,27]. In specific, Smith et al [26] found that psychological and interpersonal concerns constituted the majority of psychosocial concerns expressed by survivors across 181 online posts, with fewer references to financial issues and contextual health care delivery domains such as communication, care coordination, and care experience. Andy and Andy [27] applied LDA and Linguistic Inquiry and Word Count to 29,533 Reddit posts and identified major themes related to treatment costs, dietary changes, advice seeking, treatment and side effects, cancer-related tests, symptoms, and risk. However, these earlier approaches were limited in their ability to capture semantic nuance and within-document relationships, as topic interpretation depended heavily on word-frequency patterns and manual summarization. Our analysis provides a more fine-grained understanding of how these concerns are structured and interconnected in patient narratives, especially those that are often underrecognized in routine survivorship care. Specifically, findings from centrality analyses highlight the prominence of specific topics within the network. “Fear of cancer recurrence” and “Psychological distress” consistently rank highest across centrality metrics, underscoring their pivotal role as both frequent concerns and key bridges between weakly connected topics. This finding aligns with previous evidence that fear of cancer recurrence is highly prevalent among survivors of cancer, with reported rates as high as 73%, and is often accompanied by complex emotional experiences, including depression, sadness, grief, a sense of loss, and social isolation [28]. However, these highly prevalent issues exhibit low PMI scores with most other nodes, suggesting that while they are widely referenced across stories, their pairwise co-occurrence with any single specific topic is not disproportionately strong. In other words, fear of cancer recurrence and generalized distress operate as pervasive background stressors that impact many aspects of survivorship but do not strongly bind to particular issues at the same document level.

In contrast, the strongest association was observed between “Sexual health concerns” and “Reproductive concerns,” closely followed by links to “Quality of life impact,” consistent with previous findings [29], indicating that sexual and reproductive health stressors are multidimensional and interrelated rather than isolated. This finding is particularly relevant in the context of OHCs, which are commonly used by younger generations for health information seeking, emotional expression, and peer support. These platforms may provide a more comfortable space for discussing sensitive or hard-to-disclose concerns that are often difficult to raise in routine clinical encounters or family conversations. In line with previous research, 28% to 44% of survivors of cancer who are at the prime stage for having their own children reported moderate to severe reproductive concerns [30]. The inherent gonadotoxic impact of cancer treatments can result in long-term reproductive impairment, such as permanent infertility, premature ovarian insufficiency, or testicular failure [31,32]. Evidence has shown that the proportion of infertility diagnoses was significantly higher in young survivors of cancer, with whom diagnosed as breast cancer, leukemia, lymphoma, thyroid cancer, and melanoma exhibiting 17%‐56% higher risk of subsequent infertility compared to individuals without cancer [33]. Beyond their direct impacts on germline cells, cancer treatment may also indirectly compromise sexual health by impairing sexual function and libido. For instance, survivors of breast cancer who receive endocrine therapy often experience menopausal symptoms, such as hot flashes, night sweats, and atrophic vaginitis, which can lead to dyspareunia [34]. Additionally, poor body image following surgery (eg, mastectomy and permanent ostomy) may further diminish sexual health, which is often underrecognized in survivorship care but is critically important to the overall quality of life [34]. Moreover, “Coping strategies,” defined as cognitive and behavioral efforts to reduce or adapt to stressful conditions and associated emotional distress [35], were identified as another significant connector to “Quality of life.” This connection is both logical and crucial for informing future interventions, as individuals who rely more on proactive, problem-focused coping approaches, rather than avoidance, tend to adapt more effectively to life stressors [35]. For example, acceptance and commitment therapy, which emphasizes being “unhooked” from cognitive fusion and experiential avoidance while promoting committed, value-based actions, represents a promising solution to enhance psychological flexibility, emotional acceptance, and adaptive coping [36].

### Strengths and Limitations

This study used TopicGPT, an LLM-based framework for topic modeling, to provide a structured and efficient approach for automatically extracting semantically meaningful segments and accelerating the thematic analysis of large-scale, mixed-language qualitative health care data. The observed topic frequencies and interpretable associations further demonstrate the success of topic modeling and the reliability of the established network. Notably, this study compared the TopicGPT performance among several state-of-the-art models, validating their effectiveness in topic modeling and offering valuable insights into model selection for a specific context. However, several limitations should be acknowledged. First, the results were generated using nonreasoning LLM models to optimize time and cost, which may have impacted the quality and depth of the extracted topics. Second, interrater agreement for the human annotations was only moderate, reflecting substantial subjectivity and semantic ambiguity in the annotation process. Accordingly, the identified best-performing model should be interpreted cautiously, as its performance may vary across different topics and cultural contexts. Third, the online platforms analyzed in this study may differ in their user composition, communication norms, and moderation policies, leading to heterogeneity in the concerns expressed and potentially limiting the comparability of topics across platforms. Additionally, our analysis was restricted to posts written in English and Chinese, which may overlook perspectives from other cultural contexts and further constrain the generalizability of our findings. Finally, although a modified refinement step was applied to minimize overlaps at the same hierarchical level, cross-level overlaps persisted. To better understand the underlying mechanisms, we traced the posts and found that the models occasionally assigned generalized topics as subtopics when issues were not explicitly stated but rather embedded within or secondary to the main problem. This categorization is understandable and reasonable for identifying key issues within each post, especially in cases where a single segment was assigned multiple topics. Overall, while TopicGPT demonstrates excellent consistency, interpretability, and efficiency, it should not operate in isolation; rather, human annotators remain essential for revealing inherent meaningfulness within broader sociocultural contexts beyond surface meanings of the data and for proposing solutions that directly address key pain points.

### Conclusions

Our findings demonstrate the potential of LLM-based topic modeling for context-sensitive analysis of patient-authored narratives. We compared the performance of 3 state-of-the-art LLMs using both quantitative metrics and qualitative manual evaluation. A topic co-occurrence network was subsequently constructed based on the best-performing model, DeepSeek-V3.2. Notably, this study revealed critical, interconnected concerns related to sexual and reproductive health, inadequate social support, and reduced quality of life among patients with cancer, offering a broader view of the concerns expressed by patients with cancer. Overall, the proposed domain-adaptable pipeline provides a scalable and interpretable solution for extracting high-fidelity topics and relationships from extensive qualitative data, with potential to inform future patient-centered research and guide subsequent clinical and psychosocial investigation.

## Supplementary material

10.2196/92539 Multimedia Appendix 1 Prompts summary.

10.2196/92539 Multimedia Appendix 2 Results of topic assignment and network centrality indices.

10.2196/92539 Multimedia Appendix 3 Interactive visualizations of latent Dirichlet allocation and BERTopic.

## Abbreviations

BERT: Bidirectional Encoder Representations from Transformers
LDA: latent Dirichlet allocation
LLM: large language model
OHC: online health community
PMI: pointwise mutual information

## References

[1] Bray F, Laversanne M, Sung H (2024). Global cancer statistics 2022: GLOBOCAN estimates of incidence and mortality worldwide for 36 cancers in 185 countries. CA Cancer J Clin.

[2] (2025). Cancer stat facts: cancer of any site. National Cancer Institute. SEER.

[3] Lang-Rollin I, Berberich G (2018). Psycho-oncology. Dialogues Clin Neurosci.

[4] Huang Y, Zhan Y, Zhan Y (2025). Psychological stress on cancer progression and immunosenescence. Semin Cancer Biol.

[5] Bradford NK, McDonald FEJ, Bibby H, Kok C, Patterson P (2022). Psychological, functional and social outcomes in adolescent and young adult cancer survivors over time: a systematic review of longitudinal studies. Psychooncology.

[6] Shokri M, Klapper E, Shan J, Levitan SI Finding common patterns in domestic violence stories posted on Reddit.

[7] Pham C, Hoyle A, Sun S, Resnik P, Iyyer M TopicGPT: a prompt-based topic modeling framework.

[8] Zhao C, Chen Y LLM-powered topic modeling for discovering public mental health trends in social media.

[9] Cohen J (1960). A coefficient of agreement for nominal scales. Educ Psychol Meas.

[10] Han J (2023). Data Mining.

[11] Reimers N, Gurevych I (2019). Sentence-BERT: sentence embeddings using Siamese BERT-networks.

[12] Blei DM, Ng AY, Jordan MI (2003). Latent Dirichlet allocation. J Mach Learn Res.

[13] Grootendorst M (2022). BERTopic: neural topic modeling with a class-based TF-IDF procedure. arXiv.

[14] Röder M, Both A, Hinneburg A Exploring the space of topic coherence measures.

[15] Zerr S, Bischoff K Topic cropping: leveraging latent topics for the analysis of small corpora.

[16] Sawant S, Yu J, Pandya K, Ngan CK, Bardeli R An enhanced BERTopic framework and algorithm for improving topic coherence and diversity.

[17] Church KW, Hanks P (1990). Word association norms, mutual information, and lexicography. Comput Linguist.

[18] Fusaroli M, Polizzi S, Menestrina L (2024). Unveiling the burden of drug-induced impulsivity: a network analysis of the FDA adverse event reporting system. Drug Saf.

[19] Freeman LC (1978). Centrality in social networks conceptual clarification. Soc Networks.

[20] Barrat A, Barthélemy M, Pastor-Satorras R, Vespignani A (2004). The architecture of complex weighted networks. Proc Natl Acad Sci U S A.

[21] Newman MEJ (2003). The structure and function of complex networks. SIAM Rev.

[22] Sabidussi G (1966). The centrality of a graph. Psychometrika.

[23] Opsahl T, Agneessens F, Skvoretz J (2010). Node centrality in weighted networks: generalizing degree and shortest paths. Soc Networks.

[24] Newman MEJ (2018). The New Palgrave Dictionary of Economics.

[25] Bastian M, Heymann S, Jacomy M Gephi: an open source software for exploring and manipulating networks.

[26] Smith A, Fogarasi M, Lustberg MB, Nekhlyudov L (2022). Perspectives of adolescent and young adult cancer survivors: review of community-based discussion boards. J Cancer Surviv.

[27] Andy A, Andy U (2021). Understanding communication in an online cancer forum: content analysis study. JMIR Cancer.

[28] Simard S, Thewes B, Humphris G (2013). Fear of cancer recurrence in adult cancer survivors: a systematic review of quantitative studies. J Cancer Surviv.

[29] Vasconcelos P, Carrito ML, Quinta-Gomes AL (2024). Associations between sexual health and well-being: a systematic review. Bull World Health Organ.

[30] Xie J, Sun Q, Duan Y (2022). Reproductive concerns among adolescent and young adult cancer survivors: a scoping review of current research situations. Cancer Med.

[31] Duffin K, Mitchell RT, Brougham MFH, Hamer G, van Pelt AMM, Mulder CL (2024). Impacts of cancer therapy on male fertility: past and present. Mol Aspects Med.

[32] Rodriguez-Wallberg KA, Jiang Y, Lekberg T, Nilsson HP (2023). The late effects of cancer treatment on female fertility and the current status of fertility preservation—a narrative review. Life (Basel).

[33] Velez MP, Richardson H, Baxter NN (2021). Risk of infertility in female adolescents and young adults with cancer: a population-based cohort study. Hum Reprod.

[34] Wagle NS, Nogueira L, Devasia TP (2025). Cancer treatment and survivorship statistics, 2025. CA Cancer J Clin.

[35] Holahan CJ, Moos RH, Groesz LM (2007). Encyclopedia of Stress.

[36] Hayes SC, Luoma JB, Bond FW, Masuda A, Lillis J (2006). Acceptance and commitment therapy: model, processes and outcomes. Behav Res Ther.

