# Supplementary material for: Exploring the Narratives of Patients With Cancer Using Large Language Models: Topic Modeling and Social Network Analysis
Source: J Med Internet Res. 2026 Jul 6;28:e92539. doi: 10.2196/92539 (PMC13335942; doi:10.2196/92539)
Supplement: Multimedia Appendix 1 [file jmir-v28-e92539-s001.pdf]

Prompts Summary

Prompt 1: Top-level topic generation

You will receive a document and a set of top-level topics from a topic hierarchy. Your task is to identify generalizable topics within the document that can act as top-level topics in the hierarchy. If any relevant topics are missing from the provided set, please add them. Otherwise, output the existing top-level topics as identified in the document.

[Top-level topics]
{Topics}

[Examples]
Example 1: Adding "[1] Psychological distress"
Document:
I'm pretty depressed at this point. I just want my life back, I've forgotten who I am.

Your response:
[1] Psychological distress: Mentions a set of painful mental and physical symptoms that are associated with normal fluctuations of mood in most people.

Example 2: Duplicate "[1] Reproductive concerns", returning the existing topic
Document:
I'm nervous for many reasons, but my main concern is that I have a 3 month old baby who weighs approximately 16 pounds.

Your response:
[1] Reproductive concerns: Mentions worries and uncertainties regarding their ability to have biological children in the future, personal and children's health, acceptance of possibly not being able to have children, discussing potential fertility problems with a spouse or partner, and trying to become pregnant.

[Instructions]
Step 1: Determine topics mentioned in the document.
- The topic labels must be as GENERALIZABLE as possible. They must not be document-specific.
- The topics must reflect a SINGLE topic instead of a combination of topics.
- The new topics must have a level number, a short general label, and a topic description.
- The topics must be broad enough to accommodate future subtopics.
Step 2: Perform ONE of the following operations:
1. If there are already duplicates or relevant topics in the hierarchy, output those topics and stop here.
2. If the document contains no topic, return "None".
3. Otherwise, add your topic as a top-level topic. Stop here and output the added topic(s). DO NOT add any additional levels.

[Document]
{Document}

Please ONLY return the relevant or modified topics at the top level in the hierarchy.
[Topic Level] Topic Label: Topic Description

Your response:

Prompt 2: Subtopic generation

You will receive a branch from a topic hierarchy along with some documents assigned to the top-level topic of that branch. Your task is to identify generalizable second-level topics that can act as subtopics to the top-level topic in the provided branch. Add your topic(s) if they are missing from the provided branch. Otherwise, return the existing relevant or duplicate topics.

[Example: Return "[2] Anxiety" (new) and "[2] Depression" (existing) as the subtopics of "[1] Psychological distress" (provided).]
Topic branch:
[1] Psychological distress
    [2] Depression
    [2] Anxiety

Document 1:
although i know im lucky and it could be worse i cant help being extremely anxious now and just waiting for my body to break again.

Document 2:
I am depressed and i do not want to get out of bed.

Document 3:
I am keeping quite positive but feeling really anxious waiting for results.

Your response:
[1] Psychological distress
    [2] Anxiety (Document: 1, 3): Mentions an emotion characterized by apprehension and somatic symptoms of tension in which an individual anticipates impending danger, catastrophe, or misfortune.
    [2] Depression (Document: 2): Mentions a persistent feeling of sadness, discontent, loss of interest, pessimism, and despondency that interferes with daily life, or somatic and cognitive changes that significantly affect the individual's capacity to function.

[Instructions]
Step 1: Determine PRIMARY and GENERALIZABLE topics mentioned in the documents.
- The topics must be generalizable among the provided documents.
- Each topic must not be too specific so that it can accommodate future subtopics.
- Each topic must reflect a SINGLE topic instead of a combination of topics.
- Each top-level topic must have a level number and a short label. Second-level topics should also include the original documents associated with these topics (separated by commas) as well as a short description of the topic.
- The number of topics proposed cannot exceed the number of documents provided.
Step 2: Perform ONE of the following operations:
1. If the provided top-level topic is specific enough, DO NOT add any subtopics. Return the provided top-level topic.
2. If your topic is duplicate or relevant to the provided topics, DO NOT add any subtopics. Return the existing relevant topic.
3. If your topic is relevant to and more specific than the provided top-level topic, add your topic as a second-level topic. DO NOT add to the first or third level of the hierarchy.

[Topic branch]
{Topic}

[Documents]
{Document}

DO NOT add first- or third-level topics.

Your response:

Prompt 3: Topic refinement

You will receive a list of topics that belong to the same level of a topic hierarchy. Your task is to merge topics that are paraphrases or near duplicates of one another. Return "None" if no modification is needed.

Here are some examples:
[Example 1: Merge "[1] Emotional distress" (new) with an existing similar topic "[1] Psychological distress" (existing).]
Topic List:
[1] Emotional distress
[1] Psychological distress

Your response:
Topics being merged into an existing topic:
[1] Psychological distress

[Example 2: Return similar topics "[1] Negative body image" (new) and "[1] Appearance distress" (new) as a new top-level topic "[1] Body image distress" (new).]
[1] Negative body image
[1] Appearance distress

Your response:
Topics being merged into a new topic:
[1] Body image distress

[Example 3: Return similar topics "[2] Drug abuse" (new) and "[2] Drug addiction" (new) as a new subtopic of "[1] Substance use" (existing).]
[1] Substance use
    [2] Drug abuse
    [2] Drug addiction

Your response:
Topics being merged into a new topic:
[1] Substance use
    [2] Drug abuse

[Rules]
- Each line represents a topic, with a level indicator and a topic label.
- Perform the following operations as many times as needed:
    - Merge relevant topics into a single topic.
    - Do nothing and return "None" if no modification is needed.
- When merging, the output format should contain a level indicator, the updated label and description, followed by the original topics.

[Topic List]
{Topics}

Output the modification or "None" where appropriate. Do not output anything else.
[Your response]

Prompt 4: Topic assignment

You will receive a document and a topic hierarchy. Assign the document to the most relevant topics the hierarchy. Then, output the topic labels, assignment reasoning and supporting quotes from the document. DO NOT make up new topics or quotes.

[Topic Hierarchy]
{tree}

[Examples]
Example 1: Assign "[1] Psychological distress" to the document
Document:
I am really struggling with the fatigue, to the point it's becoming distressing. I can't function day to day, I have to force myself to go to work.

Assignment:
[1] Psychological distress: Mentions a set of painful mental and physical symptoms that are associated with normal fluctuations of mood in most people. ("...it's becoming distressing. I can't function day to day...")

Example 2: Assigned "[1] Fear of cancer recurrence" to the document
Document:
I think the thought of reoccurrence would eat me alive everyday and not let me live.

Assignment:
[1] Fear of cancer recurrence: Mentions the fear or worry that cancer could return or progress in the same place or another part of the body. ("...the thought of reoccurrence would eat me alive everyday and not let me live.")

[Instructions]
1. Topic labels must be present in the provided topic hierarchy. You MUST NOT make up new topics.
2. The quote must be taken from the document. You MUST NOT make up quotes.

[Document]
{Document}

Double check that your assignment exists in the hierarchy!
Your response should be in the following format:
[Topic Level] Topic Label: Assignment reasoning (Supporting quote)

Your response:

Prompt 5: Topic correction

You will receive a document and a topic hierarchy. Assign the document to the most relevant topics the hierarchy. Then, output the topic labels, assignment reasoning and supporting quotes from the document. DO NOT make up new topics or quotes.

[Topic Hierarchy]
{tree}

[Examples]
Example 1: Assign "[1] Psychological distress" to the document
Document:
I am really struggling with the fatigue, to the point it's becoming distressing. I can't function day to day, I have to force myself to go to work.

Assignment:
[1] Psychological distress: Mentions a set of painful mental and physical symptoms that are associated with normal fluctuations of mood in most people. ("...it's becoming distressing. I can't function day to day...")

Example 2: Assigned "[1] Fear of cancer recurrence" to the document
Document:
I think the thought of reoccurrence would eat me alive everyday and not let me live.

Assignment:
[1] Fear of cancer recurrence: Mentions the fear or worry that cancer could return or progress in the same place or another part of the body. ("...the thought of reoccurrence would eat me alive everyday and not let me live.")

[Instructions]
1. Topic labels must be present in the provided topic hierarchy. You MUST NOT make up new topics.
2. The quote must be taken from the document. You MUST NOT make up quotes.

[Document]
{Document}

{Message} Double check that your assignment exists in the hierarchy!
Your response should be in the following format:
[Topic Level] Topic Label: Assignment reasoning (Supporting quote)

Your response:

Seed Topics

[1] Psychological distress: Mentions a set of painful mental and physical symptoms that are associated with normal fluctuations of mood in most people.

[1] Fear of cancer recurrence: Mentions the fear or worry that cancer could return or progress in the same place or another part of the body.

[1] Reproductive concerns: Mentions worries and uncertainties regarding their ability to have biological children in the future, personal and children's health, acceptance of possibly not being able to have children, discussing potential fertility problems with a spouse or partner, and trying to become pregnant.

[1] Social Isolation: Mentions reduced or absence of social contact.

[1] Stigma: Mentions perceived or experienced negative social attitude, social disapproval, and discrimination.
