# Supplementary material for: Exploring the Narratives of Patients With Cancer Using Large Language Models: Topic Modeling and Social Network Analysis
Source: J Med Internet Res. 2026 Jul 6;28:e92539. doi: 10.2196/92539 (PMC13335942; doi:10.2196/92539)
Supplement: Multimedia Appendix 2 [file jmir-v28-e92539-s002.docx]

**Table S1. Example topic assignments from TopicGPT, LDA, and BERTopic on two documents.**

| **Document** | **TopicGPT** | **LDA** | **BERTopic** |
| --- | --- | --- | --- |
| "Hi All I had a smx in September for ILC. No node involvement and chemo not needed. However the oncologist has recommended 5 days of rads due to the size of the multifocal tumours (4.8cm and 1.8cm). I'm having my 3rd radiotherapy session today but I'm starting to freak out because I've read that most people who've had a mastectomy don't have radiotherapy and recurrence is more likely with radiotherapy to smx side as there's no breast tissue. I'm just scared that I've been overtreated and the long term effects of radio scares me. I'm literally gripped with fear right now…" (#2851) | Fear of cancer recurrence; Fear of treatment failure; Treatment decision concerns; Adjuvant therapy decisions; Psychological distress | Topic_12: ['surgery', 'surgeon', 'pain', 'implant', 'day', 'ago', 'nerve', 'week', 'nurse', 'month', 'eye', 'time', 'right', 'removed', 'left'] | Topic_15: ['breast', 'dcis', 'mastectomy', 'lymph', 'nodes', 'invasive', 'grade', 'cancer', 'node', 'lymph nodes', 'lumpectomy', 'breast cancer', 'chemo', 'surgery', 'lobular'] |
| "I'm 31. I was diagnosed with multiple sclerosis at 25 and really thought that had to be it for a while, right? Nope. I was diagnosed with cervical cancer December 17th. And then told that, while it's treatable, all recommended treatments will rob me of my ability to have children the day before Christmas Eve. The doctor said she doesn't want to surgically remove my 3.7cm tumor, that she doesn't recommend it for any tumor over 2cm. I don't know anything about this stuff and my support system is so small. But I'm having a very hard time accepting the loss of ever having my own family. I don't know how to go about getting a second opinion or if it's even worth it. Any advice, or words of comfort, anything would be appreciated this Christmas Eve…" (#145) | Reproductive concerns; Fertility impact; Social support; Informational support; Treatment decision concerns | Topic_11: ['anxiety', 'treatment', 'time', 'month', 'year', 'work', 'life', 'nurse', 'chemo', 'good', 'struggle', 'fear', 'blood', 'new', 'wear'] | Topic_9: ['cervical', 'cervical cancer', 'cancer', 'diagnosed', 'stage', 'diagnosed cervical', 'family', 'treatment', 'diagnosis', 'week', 'ago', 'scared', 'children', 'worried', 'kids'] |

**Table S2. Centrality indices for top 20 most influential topics**

| **Rank** | **Topics** | **Frequency** | **Topics** | **Weighted degree centrality** |  | **Closeness centrality** |  | **Betweenness centrality** |  | **Eigenvector centrality** |
| --- | --- | --- | --- | --- | --- | --- | --- | --- | --- | --- |
| 1 | Fear of cancer recurrence | 1507 | Diagnostic delays and misdiagnosis | 36.3899 | Psychological distress | 0.9167 | Fear of cancer recurrence | 95.3334 | Psychological distress | 1.0000 |
| 2 | Psychological distress | 909 | Information seeking | 27.8497 | Fear of cancer recurrence | 0.8919 | Psychological distress | 91.8334 | Fear of cancer recurrence | 0.9869 |
| 3 | Treatment decision concerns | 497 | Staging uncertainty and confusion | 23.1894 | Quality of life impact | 0.7857 | Social Isolation | 38.5589 | Quality of life impact | 0.9464 |
| 4 | Diagnostic concerns | 466 | Fatigue and weakness | 22.8699 | Treatment side effects | 0.7674 | Diagnostic delays and misdiagnosis | 22.7238 | Recovery concerns | 0.9364 |
| 5 | Treatment side effects | 441 | Patient-provider communication challenges | 21.0293 | Recovery concerns | 0.7674 | Treatment side effects | 19.2099 | Treatment side effects | 0.9033 |
| 6 | Recovery concerns | 432 | Treatment pathway uncertainty | 18.2068 | Social Isolation | 0.7500 | Quality of life impact | 16.4168 | Treatment decision concerns | 0.8918 |
| 7 | Quality of life impact | 398 | Pain and discomfort | 17.8995 | Treatment decision concerns | 0.7500 | Treatment decision concerns | 13.5453 | Social Isolation | 0.8766 |
| 8 | Fear of medical procedures | 330 | Quality of life impact | 11.2057 | Diagnostic concerns | 0.7174 | Employment concerns | 9.7715 | Diagnostic concerns | 0.8463 |
| 9 | Social isolation | 252 | Hormonal and menopausal symptoms | 10.8104 | Diagnostic delays and misdiagnosis | 0.7021 | Recovery concerns | 9.4168 | Fear of medical procedures | 0.8370 |
| 10 | Reproductive concerns | 183 | Pain management | 10.4798 | Healthcare communication | 0.7021 | Pain and discomfort | 5.8778 | Healthcare communication | 0.8211 |
| 11 | Healthcare communication | 161 | Psychological distress | 10.1723 | Fear of medical procedures | 0.7021 | Diagnostic concerns | 5.7156 | Social support | 0.7827 |
| 12 | Social support | 151 | Treatment side effects | 9.4845 | Employment concerns | 0.6735 | Healthcare communication | 4.2063 | Employment concerns | 0.7450 |
| 13 | Fear of treatment failure | 148 | Job retention challenges | 7.7876 | Social support | 0.6735 | Fatigue and weakness | 4.1960 | Diagnostic delays and misdiagnosis | 0.7039 |
| 14 | Coping strategies | 127 | Employment concerns | 7.7616 | Sleep disturbances | 0.6471 | Social support | 3.1700 | Coping strategies | 0.7000 |
| 15 | Employment concerns | 91 | Healthcare communication | 7.6546 | Coping strategies | 0.6471 | Fear of medical procedures | 2.7854 | Reproductive concerns | 0.6938 |
| 16 | Sleep disturbances | 79 | Recovery concerns | 7.4876 | Fear of treatment failure | 0.6471 | Treatment pathway uncertainty | 1.3214 | Sleep disturbances | 0.6932 |
| 17 | Treatment complications | 70 | Urinary complications | 7.3623 | Reproductive concerns | 0.6471 | Fear of treatment failure | 1.2179 | Fear of treatment failure | 0.6923 |
| 18 | Family communication concerns | 57 | Treatment complications | 7.0803 | Treatment complications | 0.6346 | Sleep disturbances | 1.0595 | Treatment complications | 0.6553 |
| 19 | Sexual health concerns | 55 | Social support | 6.7437 | Fatigue and weakness | 0.6111 | Reproductive concerns | 0.9174 | Family communication concerns | 0.5812 |
| 20 | Diagnostic delays and misdiagnosis | 50 | Sleep disturbances | 6.3970 | Family communication concerns | 0.6111 | Information seeking | 0.8111 | Sexual health concerns | 0.5358 |


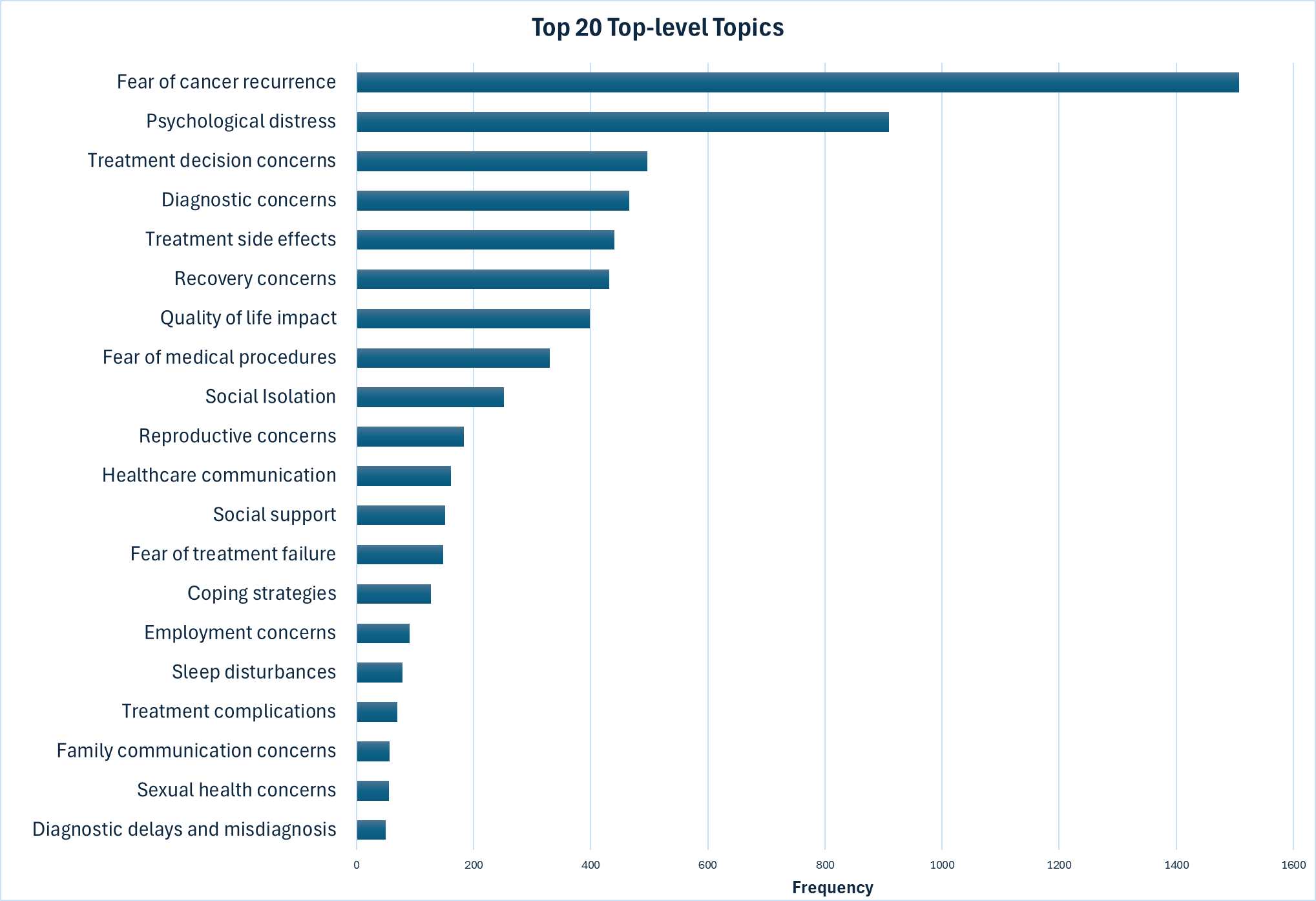


**Figure S1. The top 20 most frequent top-level topics (DeepSeek V3.2)**
