# Supplementary material for: Exploring the Narratives of Patients With Cancer Using Large Language Models: Topic Modeling and Social Network Analysis
Source: J Med Internet Res. 2026 Jul 6;28:e92539. doi: 10.2196/92539 (PMC13335942; doi:10.2196/92539)
Supplement: Multimedia Appendix 3 [file jmir-v28-e92539-s003.docx]

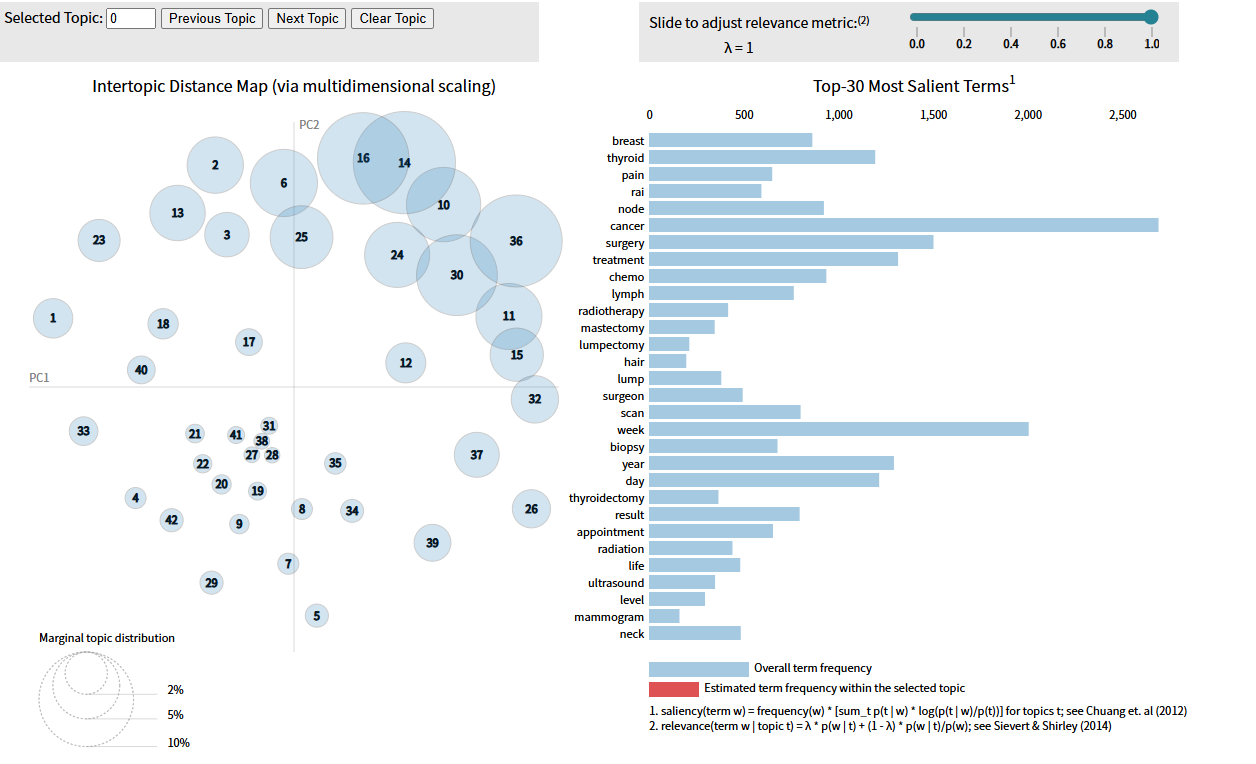


**Figure S1. The visualization of LDA model for English dataset**


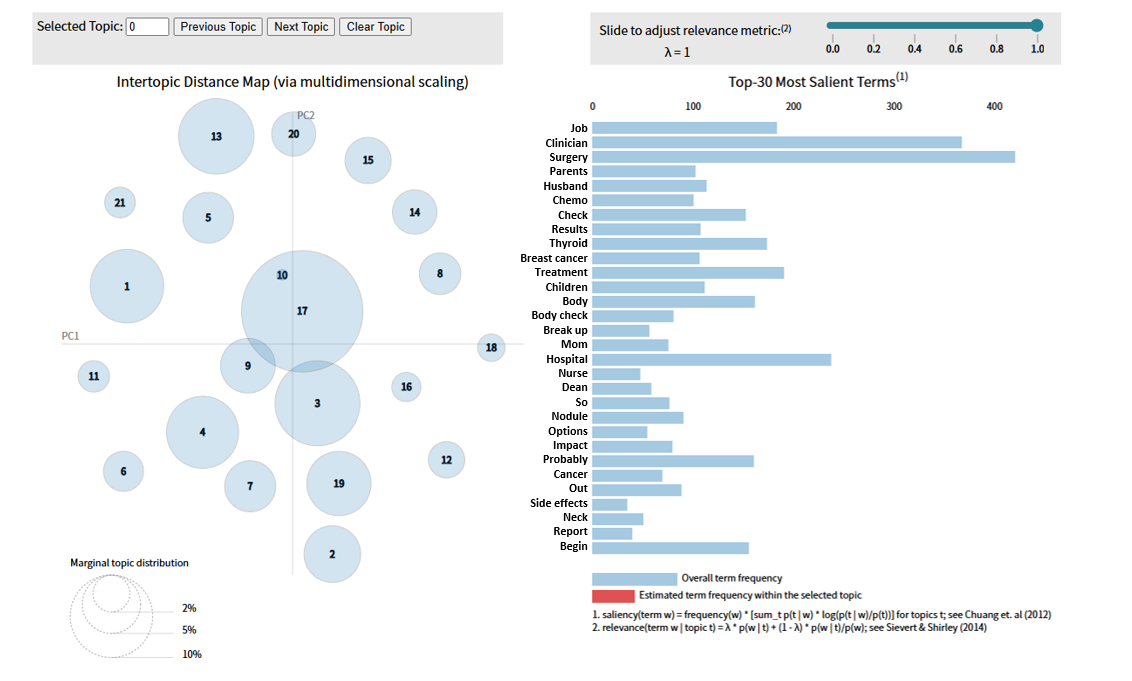


**Figure S2. The visualization of LDA model for Chinese dataset (The labels were translated from Chinese)**


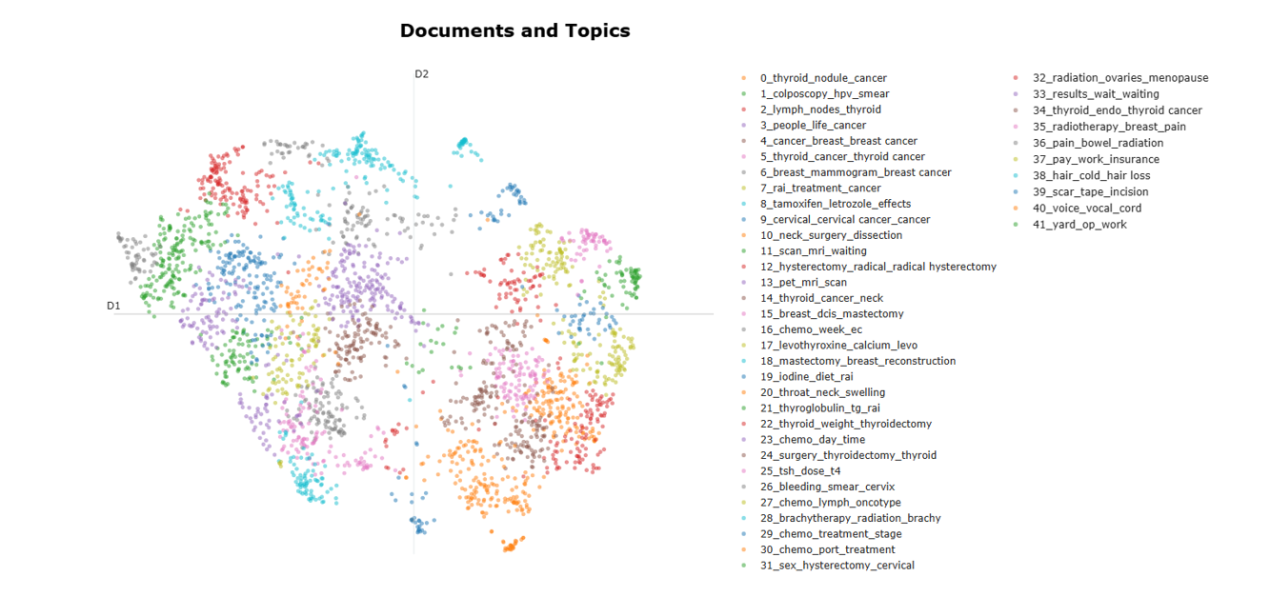


**Figure S3. The 2D UMAP document scatter plot for English dataset**


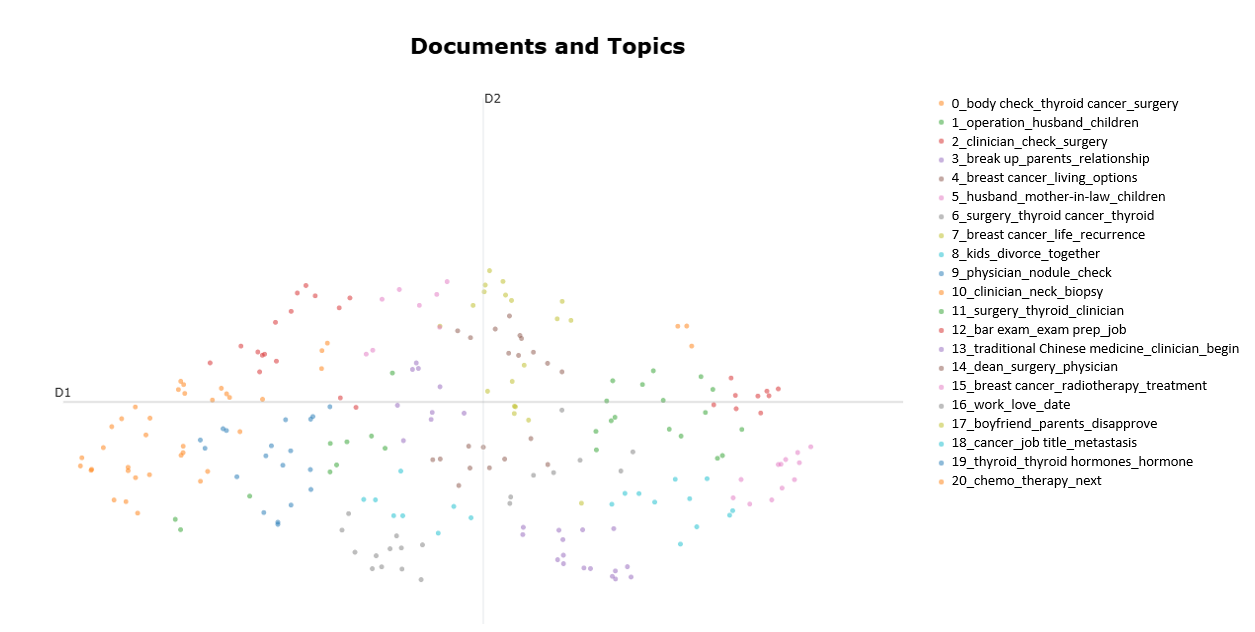


**Figure S4. The 2D UMAP document scatter plot for Chinese dataset (The labels were translated from Chinese)**
